# Biochemical Characteristics of Three Laccase Isoforms from the Basidiomycete *Pleurotus nebrodensis*

**DOI:** 10.3390/molecules21020203

**Published:** 2015-02-06

**Authors:** Xianghe Yuan, Guoting Tian, Yongchang Zhao, Liyan Zhao, Hexiang Wang, Tzi Bun Ng

**Affiliations:** 1 State Key Laboratory for Agrobiotechnology and Department of Microbiology, China Agricultural University, Beijing 100193, China; yuanxianghe1231@gmail.com; 2 Institute of Biotechnology and Germplasmic Resource, Yunnan Academy of Agricultural Science, Kunming 650223, China; tiangt@aliyun.com (G.T.); yaasmushroom@aliyun.com (Y.Z.); 3 College of Food Science and Technology, Nanjing Agricultural University, Weigang, Nanjing 210095, China; zhlychen@njau.edu.cn; 4 School of Biomedical Sciences, Faculty of Medicine, The Chinese University of Hong Kong, Shatin, New Territories, Hong Kong, China

**Keywords:** laccase isoenzymes, enzyme characterization, biochemical properties comparison, *Pleurotus nebrodensis*

## Abstract

The characterization of three laccase isoforms from *Pleurotus nebrodensis* is described. Isoenzymes Lac1, Lac2 and Lac3 were purified to homogeneity using ion exchange chromatography on DEAE-cellulose, CM-cellulose and Q-Sepharose and a gel filtration step on Superdex 75. The molecular weights of the purified laccases were estimated to be 68, 64 and 51 kDa, respectively. The isoenzymes demonstrated the same optimum pH at 3.0 but slightly different temperature optima: 50–60 °C for Lac1 and Lac3 and 60 °C for Lac2. Lac2 was always more stable than the other two isoforms and exposure to 50 °C for 120 min caused 30% loss in activity. Lac2 was relatively less stable than the other two isoforms when exposed to the pH range of 3.0–8.0 for 24 h, but inactivation only occurred initially, with around 70% residual activity being maintained during the whole process. Oxidative ability towards aromatic compounds varied substantially among the isoforms and each of them displayed preference toward some substrates. Kinetic constants (K_m_, K_cat_) were determined by using a 2,2′-azino-bis(3-ethylbenzothiazoline-6-sulfonic acid) diammonium salt (ABTS) assay, with Lac3 showing the best affinity and Lac2 displaying the highest catalytic efficiency. Amino acid sequences from peptides derived from digestion of isoenzymes showed great consistency with laccases in the databases.

## 1. Introduction

Laccases (EC 1.10.3.2, *p*-diphenol: dioxygen oxidoreductase) are copper-containing enzymes that catalyze the oxidation of a broad spectrum of phenolic compounds and non-phenolic substrates using molecular oxygen as the electron acceptor [1]. Laccases are common in Nature and exist extensively in plants, fungi, bacteria as well as insects [2]. However, the fact that a large number of oxidative enzymes exists in plants and many of them have low enzymic activity make purification of plant laccases difficult [3]. Meanwhile, only a limited number of bacterial laccases has been found [4], therefore, fungal laccases are of tremendous importance. Actually most characterized laccases are from fungi, and only fungal laccases have been put into biotechnological applications [5].

Purification requires a series of steps to remove contaminants; precipitation using ammonium sulphate, ultrafiltration, ion-exchange chromatography and size exclusion chromatography are the most frequently used techniques [4]. Most ligninolytic fungi produce at least one laccase isoenzyme and in fact, more than one isoenzyme are produced in most white-rot fungi [6]. There are several laccase genes in fungal genomes encoding isoenzymes and they have been suggested to be differentially regulated [7], meaning both constitutive and inductive forms can be produced. The well-studied white-rot fungus, *Pleurotus ostreatus*, produces at least eight different laccase isoenzymes, and six have been isolated and characterized [8,9,10]. The relative levels of isoenzymes are greatly dependent on physiological factors [1,7,11]. Copper was the most efficient inducer of *P. ostreatus* laccase activities [11], while C/N ratio, aromatic compounds and copper all influenced *Coprinus comatus* laccase isoenzyme profiles and their activities [1].

Laccases have attracted much attention from researchers during the past decades with regard to their potential to oxidize phenolic subunits of lignin, non-phenolic compounds and even some highly recalcitrant environmental pollutants [12], such as pesticides [13], polycyclic aromatic hydrocarbons [14] and chlorophenols [15]; and compared with chemical methods, enzymatic oxidation is specific, efficient and ecologically sustainable. Due to these advantages for biotechnological applications, laccases are already used in large scale in food, pharmaceutical and chemical industries and their applications in bioremediation are studied extensively [4].

*Pleurotus nebrodensis* is widely cultivated in many countries as a delicious and nutritious edible mushroom. *P. nebrodensis* is an efficient laccase producer and one isoform has been characterized [16]. The fact that laccases are complex and their biotechnological applications raise our interest to further investigate the laccase family of *P. nebrodensis*. In this study, we successfully purified three novel laccase isoenzymes showing great similarities to previous laccases. The biochemical properties of these laccase isoenzymes were tested. The aim of this work was to expand our knowledge of individual laccase isoenzymes in fungi.

## 2. Results

### 2.1. Purification of Laccases

**Figure 1:**
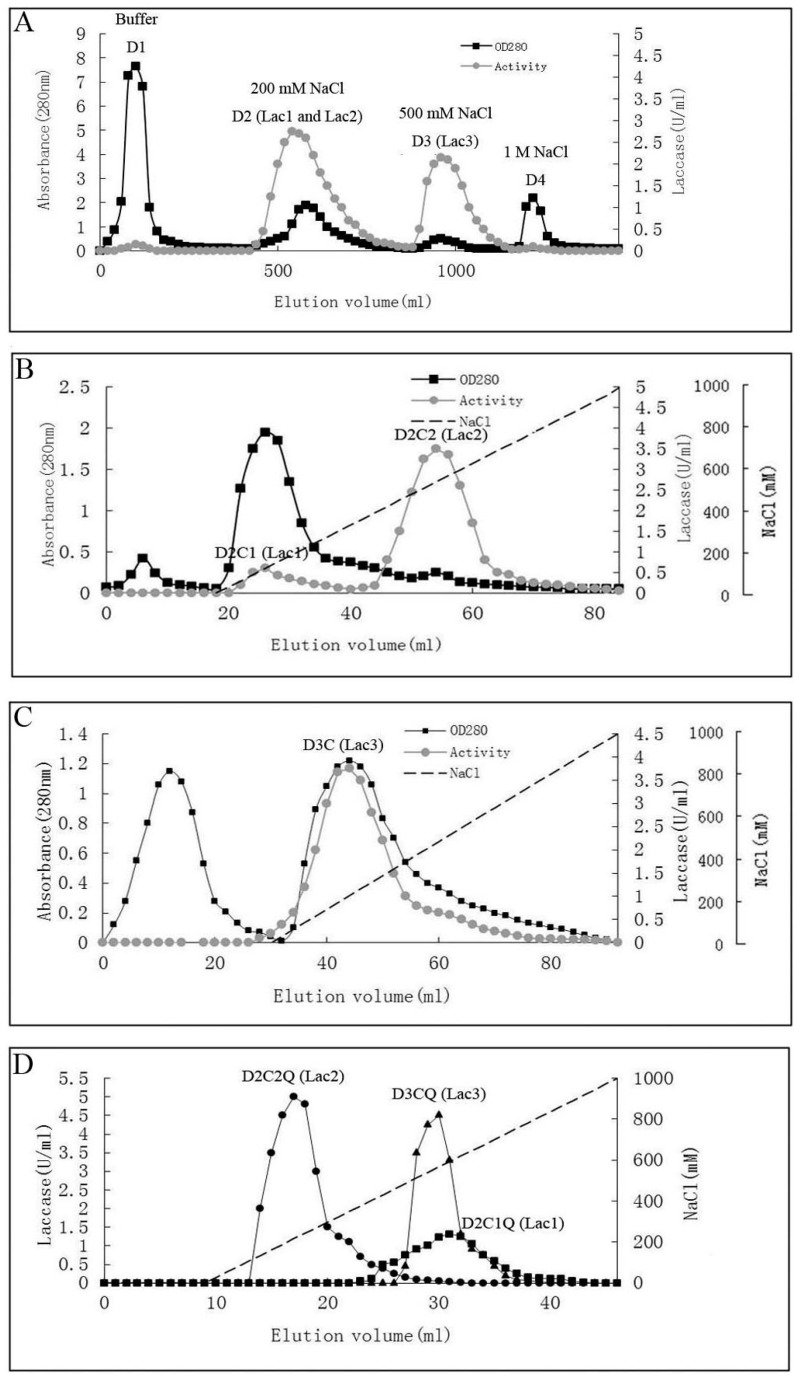
Chromatographic profiles of laccase isoforms. Black dotted line: absorbance at 280 nm; grey dotted line: activity of laccase isoforms; dashed line: NaCl gradient. (**A**) Ion exchange charomatography on DEAE-cellulose column. Lac1 and Lac2 resided in fraction D_2_ and Lac3 resided in D_3_; (**B**) Ion exchange chromatography of fraction D_2_ on CM-cellulose column. Two peaks with activity: Lac1 resided in peak D_2_C_1_ and Lac2 resided in peak D_2_C_2_; (**C**) Ion exchange chromatography of fraction D_3_ on CM-cellulose column. Lac3 resided in the only peak with activity; (**D**) Ion exchange chromatography of fractions Lac1 (D_2_C_1_), Lac2 (D_2_C_2_) and Lac3 (D_3_C) on Q-Sepharose column (showing in the same figure, results of three separate fractionation experiments for Lac1, Lac2 and Lac3, respectively).

**Figure 2:**
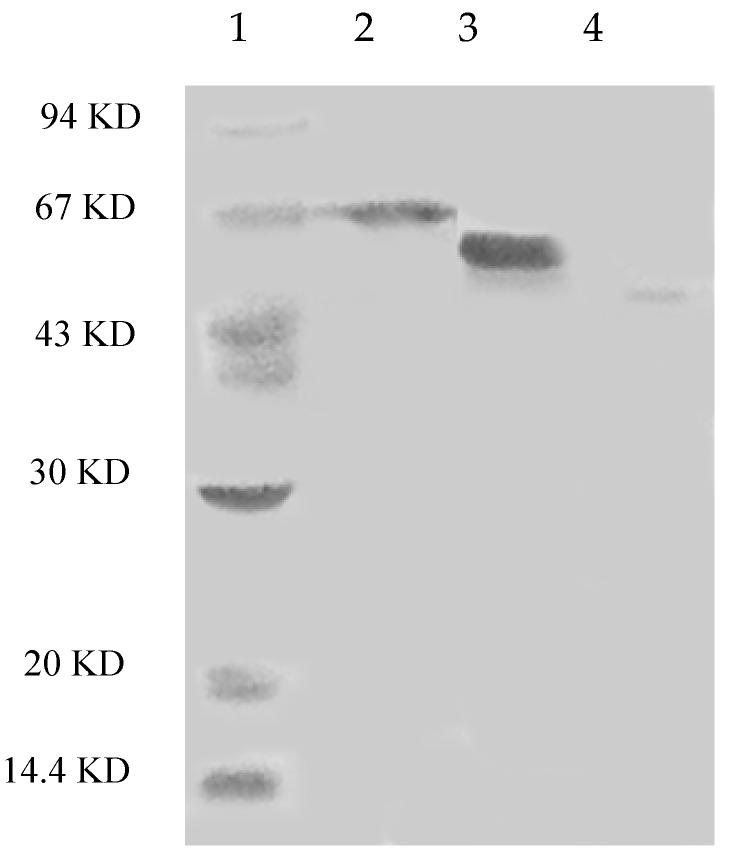
SDS-PAGE of isolated laccases. Lane 1: Molecular markers (GE Healthcare). From top to bottom: phosphorylase b (94 KD), bovine serum albumin (67 KD), ovalbumin (43 KD), carbonic anhydrase (30 KD), soybean trypsin inhibitor (20 KD) and lactalbumin (14.4 KD; Lane 2: Lac1; Lane 3: Lac2; Lane 4: Lac3).

The steps for purification and the enzyme yields are summarized in Table 1. Laccase isoforms from *P. nebrodensis* were purified by using continuous ion-exchange chromatography on DEAE-cellulose, CM-cellulose and Q-Sepharose and a final step of gel filtration by FPLC on a Superdex 75 column. All the active fractions could be adsorbed in ion-exchange chromatography. In the first step using DEAE-cellulose, a yellowish pigment D_1_ from crude extract was removed and then two fractions (D_2_ and D_3_), which were almost colorless but with strong laccase activity, were collected separately (Figure 1A). After the second step on a CM-cellulose column, two active peaks (D_2_C_1_ and D_2_C_2_) were found in the only adsorbed fraction from D_2_ by elution with a salt gradient (0–1.0 M NaCl). The second peak showed much stronger activity than that of the first peak (Figure 1B). However, there was only one active peak (D_3_C) derived from D_3_ after D_3_ had been loaded onto CM-cellulose (Figure 1C). Subsequently ion-exchange chromatography on Q-Sepharose (Figure 1D) followed by gel filtration (Superdex 75) was carried out for each isoform separately resulting in electrophoretically homogeneous preparations of three forms of laccase, which were designated as Lac1 (D_2_C_1_Q), Lac2 (D_2_C_2_Q) and Lac3 (D_3_CQ), respectively. This procedure produced 1.575 mg purified Lac1, and for Lac2 and Lac3, the yields were 1.125 mg and 0.131 mg, respectively. The fold of purification for the three isoforms was surprisingly high, attaining 55.32, 183.55 and 1378.95 fold for Lac1, Lac2 and Lac3, respectively (Table 1). Molecular weights estimated by FPLC on Superdex 75 were 70 kDa, 70 kDa and 50 kDa for Lac1, Lac2 and Lac3, respectively. Based on SDS-PAGE, the molecular weights of denatured laccases, Lac1, Lac2 and Lac3, were estimated to be 68 kDa, 64 kDa and 51 kDa, respectively (Figure 2). This indicated that these isoforms were all monomeric proteins.

**Table 1:** Summary of the results obtained during the process of purification of the three laccases from *P. nebrodensis*.

Chromatographic Method	Chromatographic Fraction by Steps	Yield (mg)	Total Activity (U) ^a^	Specific Activity (U/mg) ^b^	Recovery of Activity (%)	Purification Fold ^c^
None	Crude extract	23040	2853	0.12	100	1
DEAE-cellulose anion exchange	D_2_ (Lac 1 and Lac2)	3200	1323	0.41	46.38	3.3
	D_3_ (Lac3)	175	689	3.94	24.14	32
CM-cellulose cation exchange	D_2_C_1_ (Lac1)	62	33	0.53	1.16	4.2
	D_2_C_2_ (Lac2)	32	297	9.31	10.42	75
	D_3_C (Lac3)	29	64	2.24	2.24	18
Q-Sepharose anion exchange	D_2_C_1_Q (Lac1)	31	18.6	0.59	0.65	4.8
	D_2_C_2_Q (Lac2)	4.2	49.8	11.85	1.74	96
	D_3_CQ (Lac3)	9.6	27	2.81	0.95	23
Superdex 75 size exclusion	D_2_C_1_QS (Lac1)	1.58	10.8	6.86	0.38	55
	D_2_C_2_QS (Lac2)	1.13	25.6	22.76	0.90	183
	D_3_CQS (Lac3)	0.13	22.4	171	0.79	1379

^a^ Total activity: laccase activity (U/mL) in each step × volume (mL); ^b^ Specific activity: total activity/yield; ^c^ Purification fold: specific activity of each step/specific activity of the first step.

### 2.2. Effects of pH and Temperature on Laccase Activity and Stability

**Figure 3:**
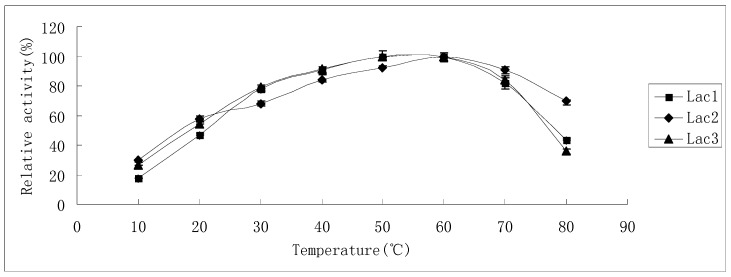
Effect of temperature on the activities of purified *P. nebrodensis* laccase isoforms. Measurements were carried out in triplicate (standard deviations for all data points < 5%).

The effect of temperature on laccase activity of Lac1 and Lac3 was similar, while Lac2 showed some differences from the other two isoforms (Figure 3). Activity increased with rise in the temperature and reached its maximum level at 50–60 °C (for Lac1 and Lac3) and 60 °C (for Lac2). When the temperature increased further, enzyme activity of Lac1 and Lac3 declined faster than that of Lac2, ensuing in only 43% and 36% of the maximum level remaining at 80 °C for Lac1 and Lac3, respectively. However, Lac2 lost only around 30% of its maximum activity at the same temperature (80 °C). Interestingly, at lower temperatures, for instance, 30 °C or 40 °C, Lac1 and Lac3 displayed a higher activity (10%) compared with Lac2 (Figure 3).

**Figure 4:**
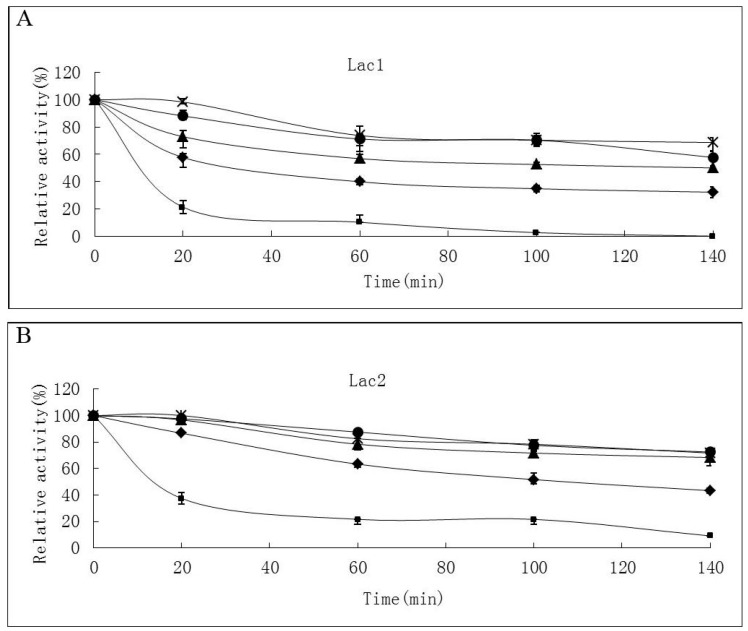

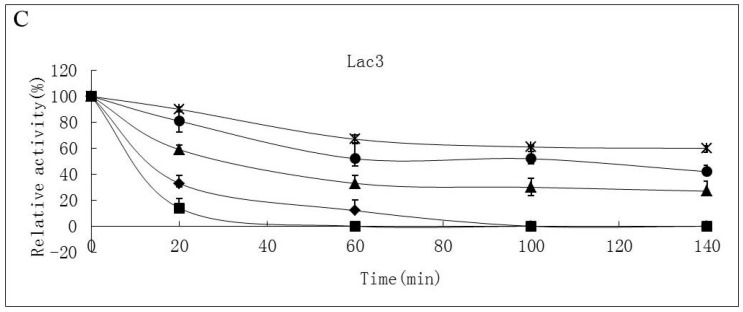
Stability of purified *P. nebrodensis* laccases at different temperatures (**A**) Effect on Lac1; (**B**) Effect on Lac2; (**C**) Effect on Lac3. “-x-” curves: treatment at 30 °C; “-●-” curves: treatment at 40 °C; “-▲-” curves: treatment at 50 °C; “-♦-” curves: treatment at 60 °C; “-■-” curves: treatment at 70 °C. Measurements were carried out in triplicate (standard deviations for all data points < 5%).

Thermostability of the laccase isoforms varied greatly. Lac2 was found to be the most thermostable and Lac1 demonstrated better thermostability compared with Lac3 in the temperature range from 30 °C to 70 °C (Figure 4A–C). After exposure Lac2 to 50 °C for 120 min, 70% of its original activity remained. A higher temperature of 70 °C, however, caused rapid inactivation (60% and 80% activity loss within 30 min and 60 min, respectively) (Figure 4B). In contrast, Lac3 was not stable even at 30 °C. Incubation for 60 min at 30 °C caused a 40% activity loss and at 50 °C, incubation for 60 min resulted in an activity loss of 70%. The enzyme was completely inactivated at 70 °C after 40 min (Figure 4C).

**Figure 5:**
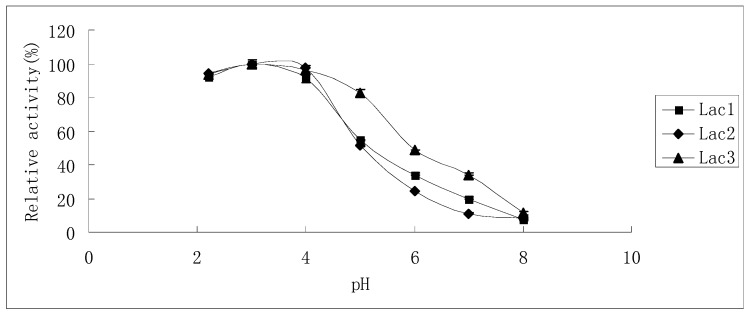
Effect of pH on the activities of purified *P. nebrodensis* laccase isoforms. Measurements were carried out in triplicate (standard deviations for all data points < 5%).

The effects of pH on laccase activity were similar among all laccase isoforms. The pH optimum for all of them was found to be around 3.0; with an increase in pH, the reaction rate declined until it became too low to be detected at pH 8.0, 7.0 and 8.0 for Lac1, Lac2 and Lac3, respectively. Besides, Lac3 had a wider activity curve compared with the other two (Figure 5).

**Figure 6:**
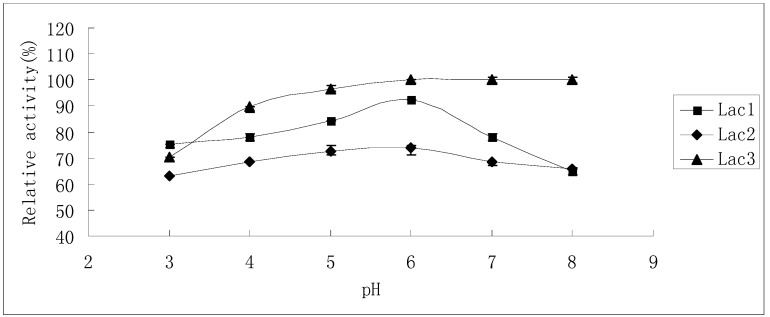
Stability of purified *P. nebrodensis* laccases after exposure to different pH values for 24 h. Measurements were carried out in triplicate (standard deviations for all data points < 5%).

The residual activities after laccase isoenzymes had been incubated in McIlvaine buffer (pH 3.0–8.0) for 24 h are shown in Figure 6. Lac3 was the most stable among the three isoforms. Preliminary results showed that Lac3 lost almost no activity when it was stored in McIlvaine buffer (pH 3.0–8.0) for 6 h (data not shown). When the incubation time was extended to 24 h, still, no obvious activity loss was observed in the range between pH 5.0 and pH 8.0 (Figure 6); though an acidic pH of 3.0 caused the greatest inactivation, the residual activity still amounted to 70% of the initial activity (Figure 6). In contrast, Lac2 was by comparison the least stable when subjected to pH values from 3.0 to 8.0; however, around 70% residual activity was detectable even after the enzyme had been treated for 24 h (Figure 6), indicating the slowing down of the inactivation process. Lac1 was more stable in acidic pH (3.0–6.0) than in neutral and alkaline pH (6.0–8.0) after treatment for a short duration. However, this enzyme was most stable at pH 6.0: exposure for 24 h brought about only 8% inactivation (Figure 6).

### 2.3. Substrate Specificity of Isolated Laccases

The specificity toward various phenolic compounds as substrates is shown in Table 2. The amount of each isoform applied in this assay was that showing the same activity to ABTS (0.05 U/mL). All the laccases readily oxidized syringaldazine and catechol, with the reactions taking place within 5 min; but virtually negligible activity was found toward l-DOPA, vanillic acid and *p*-coumaric acid, even when the reaction time was extended to 1 h. However, totally different results were obtained when the other six substrates were used. Lac3 exhibited stronger activity toward ferulic acid, caffeic acid and hydroquinone than Lac1 did (showing 27.27%, 65.45% and 75% higher activity, respectively), whereas only very weak activity could be detected when Lac2 came into contact with these substrates. Similarly, the reaction was facilitated when Lac3 was added to guaiacol or *p*-phenylenediamine, but Lac2 showed greater potential than Lac1 in oxidizing the two abovementioned substances. Intriguingly, when dimethyl phthalate was oxidized, Lac2 and Lac3 showed relatively higher activities; with Lac2 and Lac3 demonstrating comparable activities.

**Table 2:** Substrate specificities of purified *P. nebrodensis* laccases.

Substrate	Wavelength (nm)	Relative Laccase Activity (%)		
		Lac1	Lac2	Lac3
ABTS	420	100 ± 1.0	100 ± 0.0	100 ± 1.5
Guaiacol	460	16 ± 0.08	24 ± 0.08	100 ± 0.04
Dimethylphthalate	470	22.83 ± 2.17	100 ± 3.0	92.39 ± 3.26
Syringaldazine	525	100 ± 4.23	89.44 ± 4.93	84.51 ± 1.41
l-DOPA	470	0.0 ± 0.0	0.0 ± 0.0	0.0 ± 0.0
Ferulic acid	318	72.73 ± 3.64	3.64 ± 0.0	100 ± 1.82
Caffeic acid	318	34.55 ± 1.82	12.5 ± 0.0	100 ± 1.82
Vanillic acid	261	0.0 ± 0.0	0.0 ± 0.0	0.0 ± 0.0
*p*-Coumaric acid	318	0.0 ± 0.0	0.0 ± 0.0	0.0 ± 0.0
Hydroquinone	289	25 ± 1.56	6.25 ± 0.0	100 ± 3.125
Catechol	276	100 ± 0.14	92.86 ± 0.56	42.86 ± 0.28
*p*-Phenylenediamine	250	18.2 ± 0.0	54.55 ± 0.27	100 ± 0.09

Rate of substrate oxidation was determined by using the molar extinction coefficients of various substrates. The relative activity (%, mean ± standard deviation) for each substrate refers to the ratio of the activity of each laccase isoform to the maximum activity. Measurements were carried out in triplicate (standard deviations for all data points < 5%).

### 2.4. Kinetic Constants for Laccases

ABTS was used as substrate to determine the kinetic constants of purified laccases. The kinetic constants were quite different among the three purified laccases (Table 3). For example, K_m_ value of Lac2 was more than three- and four- fold higher than those of Lac1 and Lac3. However, the K_m_ value of Lac3 was somewhat lower than those of Lac1 and Lac2, indicating a slightly higher affinity of Lac3 to ABTS. Interestingly, though Lac2 showed the lowest affinity to typical substrate (ABTS), it possessed the highest K_cat_ value at the same time, suggesting a high catalytic efficiency.

**Table 3:** Kinetic constants of purified *P. nebrodensis* laccases for ABTS.

Laccase Isoforms	K_m_ (mM)	K_cat_ (min^−1^)	K_cat_/K_m_ (min^−1^·mM^−1^)
Lac1	0.16	0.958	5.99
Lac2	0.55	1.65	3.00
Lac3	0.104	0.264	2.54

### 2.5. Peptides Identified by Gel-Fractionation LC-LTQ-Orbitrap-MS

According to the data from LC-LTQ, Nine peptides, twelve peptides and three peptides obtained from Lac1, Lac2 and Lac3, respectively, closely matched previously reported laccases (Table 4). There were no identical peptides among the three purified laccases (Table 4), and Lac1, Lac2 and Lac3 showed the greatest consistency with laccases from *Pleurotus ostreatus* (gi: 15594026), *Pleurotus ostreatus* (gi: 291461620) and *Lentinus sajor-caju* (gi: 11036962), respectively (Figure S1a–c). These matched sequences exhibited high homology with other *Pleurotus* laccases but still, delicate differences such as Asp (D) in the peptide “RANPNLGSTGFDGGINSAILRY” from Lac3, made this protein different from other closest laccases (Figure S1c).

**Table 4:** Peptide sequences matching reported laccases after HPLC-LTQ-Orbitrap-MS following trypsin digestion.

	Laccaes Isoforms		
	Lac1	Lac2	Lac3
Peptides	RNDVVSPDGFERR	KVIQPDGFSRS	KGDNFQLNVVNQLSDTTMLKD
	RRAITVNGIFPGTPVILQKN	SAVLAGGSYPGPLIKG	RYAGGPTSPLAIINVESNKRY
	KNDKVQINTINELTDPGMRR	RLYDVDDESTVLTVGDWYHAPSLSLTGVPHPDSTLFNGLGRS	RANPNLGSTGFDGGINSAILRY
	RSTSIHWHGLFQHKT	SLNGPASPLYVMNVVK	
	RYKGAPAVEPTTVATTGGHKL	RYSLVLNANQAVGNYWIRA	
	RKPQDFLPSEQVIILPANKL	ANPNSGDPGFANQMNSAILRY	
	RTSNSDVVNLVNPPRR	REYNLRPLIKK	
	RDVLPINGGNTTFRF	RDAHDLAPAGSIYDIKL	
	RTLCPAYDGLAPEFQ	LGDVVEITMPALVFAGPHPLHLQWHTFAVVRS	
		SAGSSTYNYENPVRRD	
		DVVSIGDDPTDNVTIRF	

## 3. Discussion

Many laccases from *Pleurotus* spp. have been purified and characterized [10,17,18]. However, to date, there has been no study on the laccase isoenzymes from *P. nebrodensis*. Therefore, an attempt was made in the present study to demonstrate the profile of laccase isoenzymes from *P. nebrodensis*. Steps of purification led to the isolation of three homogeneous isoenzymes.

The purification techniques employed in this study, including ion exchange and gel filtration column chromatography, successfully purified three isoenzymes to homogeneity confirmed by a single laccase peak after Superdex 75 column chromatography and also single bands in SDS-PAGE gel confirm that the isoenzymes were purified to homogeneity. The purification folds (55.32, 183.55 and 1378.95 for Lac1, Lac2 and Lac3, respectively) are relatively high among laccases which have been purified from fungi [10,17,18] and bacteria [19,20]. Three purified laccases are all monomeric proteins with apparent molecular weights of 68 kDa, 64 kDa and 51 kDa. This means these molecular masses agree well with the values reported for other fungal laccases (around 50–80 kDa), e.g., *Pleurotus ostreatus* (61 kDa and 67 kDa) [10] and *Pleurotus florida* (77 kDa and 82 kDa) [17]. The zymogram analysis in this study showed three forms of laccase with partly different physico-chemical and catalytic properties, which is typical of many basidiomycetes, whereas, previous study reported only one isoform [16]. It has been shown that laccase occurs in both inducible and constitutive forms [21]. The variation in production of isoforms between different strains might be attributed to the ecological origin of the strains [22]. The C/N ratio, together with aromatic compounds and copper, significantly influenced the consitution of total laccase of *Coprinus comatus* [1]; being cultivated in high-nitrogen low carbon culturea, *Coprinus comatus* could produce six laccase isoforms, but fewer isoenzymes were detected in low-nitrogen high-carbon cultures[1]. A strain of *Pleurotus ostreatus* was also selected to investigate the effects of inducer and nitrogen concentration on laccase activity and laccase isoform patterns [23]. It’s highly likely that different strains and different culture conditions lead to the expression of more isoenzymes.

Temperature optima for Lac1 and Lac3 (50–60 °C) were slightly lower than that for Lac2 (60 °C); Lac1 and Lac3 exhibit their activities under mild conditions (<40 °C) more readily than Lac2; however, when the reaction has to proceed at higher temperatures (>55 °C), Lac2 will perform better (Figure 3). Many fungal laccases, which have been previously reported, show maximum activity at 50 °C [17,18,24,25], hence laccases reported in this study need relative higher temperatures to display their best oxidation capacity; but temperature optima were much less extreme compared with some bacterial laccases [19,26]. Lac2 could tolerate higher temperatures as compared to the other two isoforms (Figure 4A–C) and Lac1 demonstrated better thermostability than Lac3 did in the temperature range from 30 °C to 70 °C (Figure 4A–C). Lac3 was susceptible to thermal inactivation, as exposure to mild conditions (<40 °C) for only an hour engendered 50% loss in activity (Figure 4C). Because of the high thermostability and other desirable features of Lac2, this isoenzyme was selected as the purified laccase for subsequent degradation of dyes. Compared with the slight difference among temperature dependence of ABTS, the optimum pH values for ABTS were found to be all around 3.0 (Figure 5). The reaction rate reduced when pH decreased or increased. This is in consistence with the fact that fungal laccases typically exhibit pH optima for the oxidation of ABTS lower than 4.0 [6]. Lac3 was the most stable and almost no activity loss was detected when pH varied from 4.0 to 8.0 (Figure 6). The other two were relatively less stable, as inactivation became obvious once pH value was over 6.0 (Figure 6). This falls within the conclusion that the stability of fungal laccases is generally higher at acidic pH [6]. But the fact that the inactivation process for Lac2 can be slowed down offers Lac2 the possibility to be used for decolorization assay. In one word, all the laccases were stable despite with various characteristics and no drastic activity loss was found after 24-hour treatment. The results demonstrate that there is, despite the high biochemical similarity of most laccases, a diversity of physico-chemical proterties, which may not only differs from species to species but also from strain to strain within one fungal species [22].

The study of substrate specificity suggested that purified laccases can oxidize most of typical laccase substrates. All isoenzymes prefer ABTS, syringaldazine and catechol as substrates; however, virtually no discernible activity was found towards l-DOPA, vanillic acid and *p*-coumaric acid. Lac3 elicited a higher affinity than Lac1 and Lac2 did (except for syringaldazine and catechol), signifying considerable variability of substrate specificity among the laccase isoforms. Oxidation of ABTS gave rise to a cation-radical, whereas the process of oxidation of phenolic compounds is accompanied by proton release and this may help to explain why the isoforms manifest the same activity to ABTS but display a distinctly different affinity when aromatic compounds are introduced [27]. In laccases, substrate oxidation involves the “outer-sphere” mechanism in which the redox potential difference between substrate and T1 Cu site determines the electron transfer and thus, the oxidation rate [28]. Meanwhile, other factors, for instance, the composition, structure or pKa of substrates, are also known to play a pivotal role in determining the oxidation rates [29,30,31,32].

ABTS, a conventional laccase substrate, was employed to determine the kinetic constants for laccase isoforms. The three isoforms demonstrated relatively high K_m_ values compared with many other fungal laccases [33,34]. Nevertheless, when compared to some bacterial laccases [19], all three isoenzymes evinced a higher affinity toward ABTS. As the K_m_ value for Lac3 was lower than those of Lac1 and Lac2, ABTS is a better substrate for Lac3. Though Lac2 showed the lowest affinity to typical substrates (ABTS), it possessed the highest K_cat_ value at the same time, suggesting a higher catalytic efficiency. However, this catalytic efficiency was still lower than the majority of laccases [19,33,34], even though laccases in this study possessed good thermal and pH tolerance. Hopefully future studies can bring about an enhancement of the substrate affinity and catalytic efficiency by locating and modifying the substrate binding sites. Site-directed mutagenesis and fusing amino acids tags were feasible solutions to improve laccase activities and properties [35,36]. Our findings indicate some variations among laccase isoforms and the importance of these differences can only be clarified in future investigations of the catalytic mechanisms.

Many researchers deploy MALDI-TOF MS/MS to obtain short de novo sequenced peptides and partial amino acid sequence analyses were conducted to disclose homology with related laccases [19,37,38]; the ratios are comparable to the BLAST search results (from NCBI). We utilized gel-fractionation LC-LTQ-Orbitrap-MS, a similar technique, to compare sequence homology between isoenzymes purified in this study and related laccases in NCBI database. In contrast to two laccases from *Coprinus comatus*, which show 70% sequence identity with each other [35], peptides obtained from *P. nebrodensis* laccase isoforms shared no identical sequences. Meanwhile, the multiple peptides showing high consistency with laccases in the databases furnished evidence that three proteins belong to the laccase family. Nearly one-third of peptides from Lac1 and Lac2 were homologous to published laccases. Though only three peptides were detected from Lac3, they matched pretty well with many *Pleurotus* laccases. Many peptides not only exist in *Pleurotus* laccases, but also can be found in other species, for example, peptide “RKPQDFLPSEQVIILPANKL” and “RTSNSDVVNLVNPPRR” from Lac1 have pronounced homology to both *Lentinus sajor-caju* laccase (gi: 32399645) and *Hypsizygus marmoreus* laccase (gi: 166025441) (unpublished). More importantly, these two peptides are part of domain 1 and domain 2 interfaces of laccase that are responsible for interact with substrates (black bar in Figure S1a). Peptide “RTSNSDVVNLVNPPRR” is bifunctional that it is also part of type I (T1) Cu binding site and trinuclear Cu binding site (grey bar in Figure S1a).

Lac1 showed the greatest consistency with a laccase from *P. ostreatus* (gi: 15594026). The *P. ostreatus* laccase was purified from a 10-day culture instead of fruit bodies. The molecular mass of *P. ostreatus* laccase was determined as 67 kDa, which was comparable as the molecular mass of Lac1. Both *P. ostreatus* laccase and Lac1 performed their maximum activities under acid condition, with pH 3.6 and pH 3.0 as their optimum pH, respectively. Taking ABTS as substrate, 0.07 mM was determined as the K_m_ of *P. ostreatus* laccase, a value which was smaller than that of Lac1 (0.16 mM), meaning *P. ostreatus* laccase had a better affinity towards ABTS than Lac1 did; however, the K_cat_ value of *P.* *ostreatus* laccase (4.4 × 10^−6^ min^−1^) was much smaller than that of Lac1 (0.958 min^−1^), illustrating a much higher catalytic ability harbored by Lac1 [9]. For Lac2, *P. florida* laccase (gi: 3005981) was the best hit that had been fully characterized. The molecular mass (62 kDa) was similar as that of Lac2 (64 kDa), as well as their optimum pH, both exhibiting their maximum activity at pH 3.0. *P. florida* laccase had a boarder range to display maximum activity (20–50 °C), while optimum temperature of Lac2 was 50 °C to 60 °C. Compared with the best hit laccase, Lac2 exhibited less affinity but better catalytic ability: K_m_ and K_cat_ were 0.55 mM and 1.65 min^−1^ for Lac2, and 0.37 mM and 9 × 10^−4^ min^−1^ were determined as K_m_ and K_cat_ for *P. florida* laccase, respectively [8].

Peptides obtained from Lac3 also showed good consistency with laccases in the databases. However, there has been hardly report about the characterization about the best hits of Lac3. One report was about the recombinant laccase precursor of *P. eryngii* (gi: 56384217). The molecular mass of *P. eryngii* laccase precursor was estimated to be 58 kDa, which was larger than Lac3 (51 kDa). Both *P. eryngii* laccase precusor and Lac3 showed higher rates at acid condition, but in terms of pH stability, Lac3 was more stable across the pH range between pH 3.0 to pH 8.0. Like *P. eryngii* laccase precusor, laccase activity was remained well at 30 °C, but a temperature more than 45 °C caused more than 50% activity loss [39].

## 4. Experimental Section

### 4.1. Chemical Reagents and Microorganism

The *P. nebrodensis* strain was obtained from Agricultural Culture Collection of China (ACCC, Beijing, China), with “ACCC 50867” as collection number. Fruit bodies were cultured and purchased from Green Resource (Beijing) Organic Agricultural Technology Development Co., Ltd., Beijing, China. DEAE-cellulose and CM-cellulose were obtained from Sigma Chemical Co. (St. Louis, MO, USA). Q-Sepharose, Superdex 75 HR 10/30 and AKTA Purifier were from GE Healthcare (Wauwatosa, WI, USA). All other chemicals used were of analytical grade unless otherwise stated.

### 4.2. Enzyme Activity Assay

Laccase activity was routinely assayed using 2, 2′-azino-bis (3-ethylbenzothiazoline-6-sulfonic acid) diammonium salt (ABTS; Sigma) as the substrate. The reaction mixture for the standard assay contained 1 mM ABTS, McIlvaine buffer (pH 4.0), and the enzyme solution in a total volume of 200 μL. After incubation at 30 °C for 15 min, the reaction was stopped by adding 200 μL 5% trichloroacetic acid. The formation of the cation radical was detected by measuring the absorbance increase at 420 nm (ε_420_ = 36,000 M^−1^·cm^−1^). One unit of laccase activity was defined as the amount of enzyme that catalyzed the oxidation of 1 μmol of ABTS in 200 μL reaction mixture at 30 °C in 1 min.

### 4.3. Purification of Laccase isoforms

A water extract of *P. nebrodensis* fruit bodies (500 g) was prepared by homogenizing them in distilled water (1:4, *w*/*v*) at 4 °C overnight, followed by centrifugation at 12,000 *g* for 15 min. Subsequently, the supernatant obtained was subjected to anion exchange chromatography on a 2.5 cm × 20 cm DEAE-cellulose column (Sigma) previously equilibrated in 10 mM Tris-HCl buffer (pH 7.4), yielding a flowthrough fraction (D_1_) and three adsorbed fractions D_2_, D_3_ and D_4_, eluted with 0.2 M, 0.5 M and 1.0 M NaCl in the same Tris-HCl buffer, respectively. Using the enzyme assay described above, fraction D_2_ and D_3_ which contained enzyme activity were collected after dialysis against distilled water, and then fraction D_2_ and fraction D_3_ were further purified separately. D_2_ was loaded on a column of CM-cellulose (1.5 cm × 20 cm) which had previously been equilibrated with 10 mM HAc-NaAc (acetic acid/sodium acetate) buffer (pH 4.0). After removing unadsorbed fraction with the same buffer, adsorbed materials were eluted with a gradient of NaCl. Two fractions with activity, D_2_C_1_ and D_2_C_2_, were eluted separately. D_3_ was further purified in a similar way as D_2_, only using a smaller column (1.5 cm × 10 cm). One eluted fraction (D_3_C) showed laccase activity. After all three active fractions (D_2_C_1_, D_2_C_2_ and D_3_C) had been dialyzed, they were passed through another anion exchange column (Q-Sepharose, 1 cm × 10 cm) that had previously been equilibrated with 10 mM Tris-HCl buffer (pH 7.0). After elution with a NaCl gradient, the three active proteins (D_2_C_1_Q, D_2_C_2_Q and D_3_CQ) were subjected to gel filtration by fast protein liquid chromatography (FPLC) on a Superdex 75 HR 10/30 column using an AKTA Purifier (GE Healthcare) in 0.2 M NH_4_HCO_3_ buffer (pH 8.5).

### 4.4. Biochemical Characterization of the Purified Laccase Isoforms

#### 4.4.1. Effects of Temperature and pH on Laccase Activity and Stability

Temperature optimum and stability of each purified laccase were determined by using ABTS as substrate in McIlvaine buffer (pH 4.0). The effects of temperature on their activity were determined at different temperatures (10–80 °C). For determination of thermal stability, laccases were incubated for 2 h at different temperatures (30–70 °C); then the residual activities were measured. To determine the pH optima of the isoforms, a series of ABTS solution (1 mM) in McIlvaine buffers were used; the pH of McIlvaine buffer was changed in the pH range 2.2–8.0; pH stability was tested by storing the purified enzymes for 24 h at pH 3.0 to 8.0 in McIlvaine buffers. The optimum temperature was used as the control to determine the effect of pH on laccases, and the optimum pH was employed when searching the optimum temperature. For the temperature or pH stability experiments, the test system without manipulations at these conditions was used as the control.

#### 4.4.2. Assay for Substrate Specificity

Substrate specificity was studied using ABTS, guaiacol, dimethylphthalate (DMP), syringaldazine, l-DOPA, ferulic acid, caffeic acid, vanillic acid, *p*-coumaric acid, hydroquinone, catechol and *p*-phenylenediamine as substrates. The reaction mixture contained 5 mM substrate in 10 mM sodium acetate buffer (pH 4.0). Reaction was started by adding 10 μL enzyme solution to the mixture. According to molar extinction coefficient (ε) obtained from the literature, substrate oxidation rates were tested by measuring the absorbance change.

#### 4.4.3. Kinetic Constants of Laccase Isoforms

Michaelis-Menten constants (K_m_) and catalytic constants (K_cat_) of purified laccases were determined with various ABTS concentrations (125 μM–5000 μM) in McIlvaine buffer at the optimum pH of each isoform. Lineweaver-Burke plots were drawn from the initial rates obtained at various substrate concentrations while the amount of enzyme was kept constant.

### 4.5. Molecular Weight Determination and Peptide Identification with Gel-Fractionation LC-LTQ-Orbitrap-MS

Denaturing sodium dodecyl sulfate-polyacrylamide gel electrophoresis (SDS–PAGE) was carried out on vertical polyacrylamide slab gel as described by Laemmli and Favre [40], using a 12% separating gel and a 5% stacking gel. The buffer solutions for the separating gel and stacking gel were 0.5 M Tris-HCl (pH 8.8) and 1.5 M Tris-HCl (pH 6.8), respectively. Marker proteins (10 μL) were loaded onto the gel and subjected to electrophoresis in Tris-glycine buffer (pH 8.3) at 180 V. At the end of electrophoresis, the gel was stained with 0.1% Coomassie Brilliant Blue R-250. lgM_r_-migrate rate curve was then obtained based on the SDS-PAGE standard molecular mass marker proteins. Besides, FPLC gel filtration was previously performed using a Superdex 75 column (GE Healthcare); by using the elution volume of FPLC molecular mass markers, an elution volume-lgMr curve could be obtained. Based on these two curves, the molecular weights of the three purified laccase isoforms were calculated.

The single band of each laccase was then excised. Firstly a rapid standard SDS-PAGE step was used for protein pre-fractionation, and then detergents and other contaminants were removed. After protein extraction, trypsin digestion and peptide desalting, sequences of peptides were identified using a nano HPLC system coupled to a LTQ-Orbitrap mass spectrometer. Original data were transformed by BIWORKS and then a search through zooepidemicus database (including 10, 317 peptides) was conducted to determine the kind of protein each peptide belonged to. Searching indexes included mass values, fixed modifications, variable modifications, maximum missed cleavages, significance threshold and so on. Meanwhile, the peptides obtained by LTQ-MS could indicate the homology between the target laccase and laccases in the databases.

## 5. Conclusions

In this study, three monomeric laccase isoenzymes were isolated from fruit bodies of *P. nebrodensis*. Peptides from LC-LTQ furnished evidence that three proteins belong to the laccase family. The three laccase isoforms shared no identical peptide sequences although they showed similar physicochemical properties. Lac2 was the dominant laccase isoform and was thermostable as well as pH-stable.

## Supplementary Materials

Supplementary materials can be accessed at: http://www.mdpi.com/1420-3049/21/2/203/s1.
