# Supplementary material for: Biochemical Characteristics of Three Laccase Isoforms from the Basidiomycete Pleurotus nebrodensis
Source: Molecules. 2015 Feb 6;21(2):203. doi: 10.3390/molecules21020203 (PMC6273344; doi:10.3390/molecules21020203)
Supplement: Supplementary file 1 [file molecules-21-00203-s001.pdf]

# Supplementary Materials: Biochemical Characteristics of Three Laccase Isoforms from the Basidiomycete *Pleurotus nebrodensis*

**Xianghe Yuan, Guoting Tian, Yongchang Zhao, Liyan Zhao, Hexiang Wang and Tzi Bun Ng**

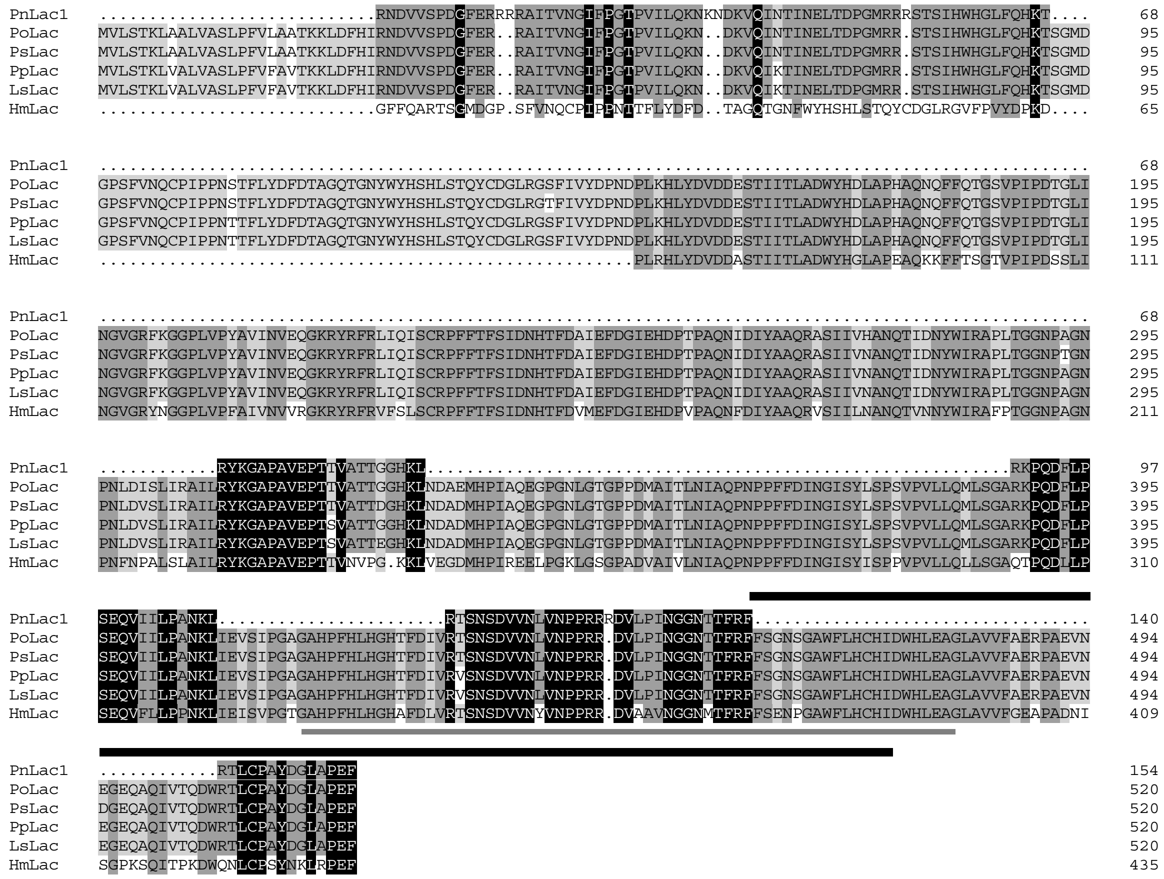


(**a**)

**Figure S1.** *Cont.*

```
PnLac2   ...................................KVIQPDGFSRSSAVLAGG..........SYPGPLIKG............................   27
PoLac    ......................MSIGPRGTLNIANKVIQPDGFSR.SAVLAGG..........SYPGPLIKGKTGDRFQINVVNKLADTSMPVDTSIHWH   67
PfLac    ..MAVAFVALVSLALALVR.VEASIGPRGTLNIANKVIQPDGFYR.STVLAGG..........SYPGPLIKGKTGDRFQINVVNKLADTSMPVDTSIHWH   86
PeLacp1  ..MAVAFIALVSLALALVR.VEASIGPRGTLNIANEVIKPDGFSR.SAVLAGG..........SYPGPLIKGETGDRFQINVVNKLADTSMPVDTSIHWH   86
PoLacp   ....................................................................................................    0
PeLacp2  MFPGARILATLTLALHLLHGTHAAIGPIADMYIVNEDVSPDGFARSAVVARSVPATDPTPASVSVPGVLVQGNKGDNFQLNVRNQLSDSTMLKTTSIHWH  100

PnLac2   ....................................................................RLYDVDDESTVLTVGDWYHAPSLSLTGVPHPD   59
PoLac    GLFVKGHNWADGPAMVTQCPIVPGHSFLYDFEVPDQAGTFWYHSHLGTQYCDGLRGPLVVYSKNDPHKRLYDVDDESTVLTVGDWYHAPSLSLTGVPHPD  167
PfLac    GLFVKGHNWADGPAMVTQCPIVPGHSFLYDFEVPDQAGTFWYHSHLGTQYCDGLRGPLVVYSKNDPHKRLYDVDDESTVLTVGDWYHAPSLSLTGVPHPD  186
PeLacp1  GIFVRGHNWADGPAMVTQCPIVPGHSFLYDFEIPDQAGTFWYHSHLGTQYCDGLRGPFVVYSKNDPHKRLYDVDDESTVLTVGDWYHAPSLSLSGVPHPD  186
PoLacp   ....................................................................REYNLR...PIIKK....PAP.....GKPFPG   20
PeLacp2  GFFQSGSTWADGPAFVNQCPIASGNSFLYDFNVPDQAGTFWYHSHLSTQYCDGLRGPFVVYDPSDPHLSLYDVDNADTVITLEDWYHVAAPQGAVLPTPD  200

PnLac2   STLFNGLGRSSLNGPASPLYVMNVVK..................................................RYSLVLNANQAVGNYWIRAANPNS  109
PoLac    STLFNGLGR.SLNGPASPLYVMNVVKGKRYRIRLINTSCDSNYQFSIDGHTFTVIEADGENTQPLQVDQVQIFAGQRYSLVLNANQAVGNYWIR.ANPNS  265
PfLac    STLFNGLGR.SLNGPASPLYVMNVVKGKRYRIRLINTSCDSNYQFSIDGHTFTVIEADGENTQPLQVDQVQIFAGQRYSLVLNANQAVGNYWIR.ANPNS  284
PeLacp1  STLFNGLGR.SLNGPASPLYVMNVVKGKRYRIRLINTSCDSNYQFSIDGHAFTVIEADGENTQPLQVDQVQIFAGQRYSLVLNANQAVGNYWIR.ANPNS  284
PoLacp   GADHN.INLNFAFDPATALFTANNHT............................................................FVP...........   48
PeLacp2  STLINGKGR.FAGGPTSPLAIINVESNKRYRFRLISMSCDPNFTFSIDGHSLQVIEADAVNIVPIVVDSIQIFAGQRYSFVLNANQAVDNYWIR.ANPNL  298

PnLac2   GDPGFANQMNSAILRYR...................EYNLRPLIKK..................................................RDAH  140
PoLac    GDPGFANQMNSAILRYKGARSIDPTTPEQNATNPLREYNLRPLIKKPAPGKPFPGGADHNINLNFAFDPATALFTANNFTFVPPTVPVLLQILSGTRDAH  365
PfLac    GDPGFENQMNSAILRYKGARSIDPTTPEQNATNPLHEYNLRPLIKKPAPGKPFPGGADHNINLNFAFDPATALFTANNHTFVPPTVPVLLQILSGTRDAH  384
PeLacp1  GDPGFANQMNSAILRYKGARNVDPTTPERNATNPLREYNLRPLIKEPAPGKPFPGGADHNINLNFAFDPATVLFTANNYTFVPPTVPVLLQILSGTRDAH  384
PoLacp   ....HTVPVLLQILS...............................................................................GTRDAH   65
PeLacp2  GSTGFEGGINSAILRYAGATENDPATTS.STSTPLLETNLVPLENPGAPGPAVPGGADININLAMGFDFTNFEMTINGSPFKAPTAPVLLQILSGATPAA  397

PnLac2   DLAPAGSIYDIKLLGDVVEITMPALVFAGPHPLHLQWHTFAVVRSSAGSSTYNYENPVRRDDVVSIGDDPTDNVTIRF.....GPFVVYSK.........  226
PoLac    DLAPAGSIYDIK.LGDVVEITMPALVFAGPHPLHLQWHTFAVVR.SAGSSTYNYENPVRR.DVVSIGDDPTDNVTIRFVADNAGPWFLHCHIDWHLDLGF  462
PfLac    DLAPAGSIYDIK.LGDVVEITMPALVFAGPHPIHLHGHTFAVVR.SAGSSTYNYENPVRR.DVVSIGDDPTDNVTIRFVADNAGPWFLHCHIDWHLDLGF  481
PeLacp1  DLAPAGSIYDIK.LGDVVEVTMPALVFAGPHPMHLHGHSFAVVR.SAGSSTYNYENPVRR.DVVSIGDDPTDNVTIRFVADNAGPWFLHCHIDWHLDLGF  481
PoLacp   DLAPAGSIYDIK.LGDVVEITMPALVFAGPHPIHLHGHTFAVVR.SAGSSTYNYENPVRR.DVVSIGDDPTDNVTIRFVADNAGPWFLHCHIDWHLDLGF  162
PeLacp2  SLLPSGSIYALA.ANKVVEISIPALAVGGPHPFHLHGHTFDVIR.SAGSTTYNFDTPARR.DVVNTGIDANDNVTIRFVTDNPGPWFLHCHIDWHLEIGL  494

PnLac2   ...................................................  226
PoLac    AVVFAEGVNQTAVANPVPEAWNNLCPIYNSSNPSKLLMGTNAIGRLPAPLK  513
PfLac    AVVFAEGVNQTAAANPVPEAWNNLCPIYNSSNPSKLLMGTNAIGRLPAPLK  532
PeLacp1  AVVFAEGVNQTAVANPVPEAWNDLCPIYNSSNPSKLLMGTNAIGRLPAPLK  532
PoLacp   AVVF...............................................  166
PeLacp2  AVVFAEDVASIKAP...PAAWDDLCPIYDALSDSDRGGIA...........  531
```

(**b**)

**Figure S1.** *Cont.*

```
PnLac3   .........................................................................KGDNFQLNVVNQLSDTTMLKD......    21
LsLac    MFPGARILATLTLALHLLHGTHAAIGPAGNMYIVNEDVSPDSFARSAVVARSVPATDPTPATASIPGVLVQGNKGDNFQLNVVNQLSDTTMLRTTSIHWH   100
PsLac    MFPGARILATLTLALHLLRGTHAAIGPIDDMYIVNEDVSPDGFTRSAVVARSVPATGPAPATVSIPGVLVQGNKGDNFQLNVVNQLSDTTMLKTTSIHWH   100
PeLac    MFPGARILATLTLALHLLHGTHAAIGPIADMYIVNEDVSPDGFARSAVVARSVPATDPTPASVSVPGVLVQGNKGDNFQLNVRNQLSDSTMLKTTSIHWH   100
PoLac    MFPGARILATLTLALHLLHGTHAAIGPIADMYIVNEDVSPDGFARSAVVARSVPATDPTPASVSVPGVLVQGNKGDNFQLNVRNQLSDSTMLKTTSIHWH   100
PpLac    MFPGARILATLTLALHLLHGALAAIGPVGDMYIVNEDVSPDGFSRSAVVARSVPITGPTPATASIPGVLVQGNKGDNFQLNVVNQLSDTTMLKTTSIHWH   100

PnLac3   ....................................................................................................    21
LsLac    GFFQAGSSWADGPAFVTQCPVASGDSFLYNFNVPDQAGTFWYHSHLSTQYCDGLRGPFVVYDPSDPHLSLYDIDNADTVITLEDWYHIVAPQNAAIPTPD   200
PsLac    GFFQFGTSWADGPAFVTQCPIASGDSFLYNFNVPDQAGTFWYHSHLSTQYCDGLRGPFVVYDPSDPHLSLYDIDNADTVITLEDWYHVVAPQNAVLPTPD   200
PeLac    GFFQSGSTWADGPAFVNQCPIASGNSFLYDFNVPDQAGTFWYHSHLSTQYCDGLRGPFVVYDPSDPHLSLYDVDNADTVITLEDWYHVAAPQGAVLPTPD   200
PoLac    GFFQSGSTWADGPAFVNQCPIAPGNSFLYDFNVPDQAGTFWYHSHLSTQYCDGLRGPFVVYDPSDPHLSLYDVDNADTVITLEDWYHVAAPQGAVLPTPD   200
PpLac    GFFQAGSSWADGPAFVTQCPVASGDSFLYNFNVPDQAGTFWYHSHLSTQYCDGLRGPFVVYDPTDPHLSLYDIDNADTVITLEDWYHIVAPQNAAKSHPD   200

PnLac3   ........RYAGGPTSPLAIINVESNKRY...............................................................RANPNLGS    50
LsLac    STLINGKGRYAGGPTSPLAIINVESNKRYRFRLVSMSCDPNFTFSIDGHSLLVIEADAVNIVPITVDSIQIFAGQRYSFVLTANQAVDKYWIRANPNLGS   300
PsLac    STLINGKSRYAGGPTSPLAIINVESTKRYRFRLVSMSCDPNFMFSIDGHSLQVIEADAVNIVPITVDSIQIFAGQRYSFVLTADQTVGNYWIRANPNLGS   300
PeLac    STLINGKGRFAGGPTSPLAIINVESNKRYRFRLISMSCDPNFTFSIDGHSLQVIEADAVNIVPIVVDSIQIFAGQRYSFVLNANQAVDNYWIRANPNLGS   300
PoLac    STLINGKGRFAGGPTSPLAIINVESNKRYRFRLISMSCDPNFTFSIDGHSLQVIEADAVNIVPIVVDSIQIFAGQRYSFVLNANQAVDNYWIRANPNLGS   300
PpLac    STLINGKGRYAGGPTSPLAVINVESTKRYRFRLVSMSCDPNFTFSIDGHSLLVIEADAVNIVPITVDSIQIFAGQRYSFVLTADQTPDNYWIRANPNLGS   300

PnLac3   TGFDGGINSAILRY......................................................................................    64
LsLac    TGFVGGINSAILRYAGATEDDPTTTSSTSTPLLETNLVPLENPGAPGPPVPGGADININLAMAFDFTTFELTINGVPFLPPTAPVLLQILSGASTAASLL   400
PsLac    TGFAGGINSAILRYAGATEDDPTTTSSTSTPLLETNLVPLENPGAPGPAVPGGADININPAMAFDFTNFELTINGAPFHAPTAPVLLQILSGATTAASLL   400
PeLac    TGFEGGINSAILRYAGATENDPATTSSTSTPLLETNLVPLENPGAPGPAVPGGADININLAMGFDFTNFEMTINGSPFKAPTAPVLLQILSGATPAASLL   400
PoLac    TGFEGGINSAILRYAGATENDPATTSSTSTPLLETNLVPLENPGAPGPAVPGGADININLAMGFDFTNFEMTINGSPFKAPTAPVLLQILSGATPAASLL   400
PpLac    TGFAGGINSAILRYVGAADADPTTTSSTSTPLLETNLVPLENPGAPGPAVPGGADININLAMAFDFTTFELTINGVPFHPPTAPVLLQILSGASSAASLL   400

PnLac3   ....................................................................................................    64
LsLac    PSGSIYELEANKVVEISMPALAVGGPHPFHLHGHTFDVIRSAGSTTYNFDTPARRDVVNTGTGAN...................................   465
PsLac    PSGSIYELEANKVVEISIPALAVGGPHPFHLHGHTFDVIRSAGSTTYNFDTPARRDVVNTGTGANDNVTIRFVTDNPGPWFLHCHIDWHLEIGLAVVFAE   500
PeLac    PSGSIYALAANKVVEISIPALAVGGPHPFHLHGHTFDVIRSAGSTTYNFDTPARRDVVNTGIDANDNVTIRFVTDNPGPWFLHCHIDWHLEIGLAVVFAE   500
PoLac    PSGSIYALEANKVVEISIPALAVGGPHPFHLHGHTFDVIRSAGSTTYNFDTPVRRDVVNTGIDANDNVTIRFVTDNPGPWFLHC........GLAVVFAE   492
PpLac    PSGSIYELAPNKVVEISMPALAVGGPHPFHLHGHTFDVIRSAGSTTYNFDTPARRDVVNTGTGANDNVTIRFVTDNPGPWFLHCHIDWHLEIGLAVVFAE   500

PnLac3   ...............................                                                                     64
LsLac    ...............................                                                                     465
PsLac    DVTSISAPPAAWDDLCPIYDALSDSDKGGIV                                                                     531
PeLac    DVASIKAPPAAWDDLCPIYDALSDSDRGGIA                                                                     531
PoLac    DVASIKAPPAAWDDLCPIYDALSDSDRGGIA                                                                     523
PpLac    DVTSISAPPAAWDALCPIYDALSDTDKGGII                                                                     531
```

(**c**)

**Figure S1.** Alignments of the amino acid sequences between each isoenzyme of *P. nebrodensis* and their related laccases. Amino acid residues that are conserved in all proteins are shaded *in black*. Residues with conserved substitutions and semiconserved substitutions are indicated with *dark grey* and *light grey*, respectively. (**a**) Alignment of the amino acid sequences between PnLac 1 and some laccases. PnLac1 from *P. nebrodensis* (Lac1); PoLac from *P. ostreatus* (gi: 15594026); PsLac from *P. sapidus* (gi: 67508841); PpLac from *P. pulmonarius* (gi: 61224796); LsLac from *Lentinus sajor-caju* (gi: 32399645); HmLac from *Hypsizygus marmoreus* (gi: 166025441). Type I (T1) Cu binding site and trinuclear Cu binding site are labeled by grey bar at bottom. Domain 1 and domain 2 interfaces of laccases are labeled by black bar at bottom; (**b**) Alignment of the amino acid sequences between PnLac2 and some Pleurotus laccases. PnLac2 from *P. nebrodensis* (Lac2); PoLac from *P. ostreatus* (gi: 291461620); PfLac from *P. florida* (gi: 3005981); PeLacp1 (a laccase precusur) from *P. eryngii* (gi: 78192166); PoLacp (a partial laccase) from *P. ostreatus* (gi: 15594026); PeLacp2 (another laccase precusur) from *P. eryngii* (gi: 56384217); (**c**) Alignment of the amino acid sequences between PnLac 3 and some laccases. PnLac3 from *P. nebrodensis* (Lac3); LsLac from *Lentinus sajor-caju* (gi: 11036962); PsLac from *P. sapidus* (gi: 50724580); PeLac from *P. eryngii* (gi: 56384217); PoLac from *P. ostreatus* (gi: 515019271); PpLac from *P. pulmonarius* (gi: 61224798).
